# Replicating an effective VA program to train and support family caregivers: a hybrid type III effectiveness-implementation design

**DOI:** 10.1186/s12913-021-06448-7

**Published:** 2021-05-06

**Authors:** N. A. Boucher, L. L. Zullig, M. Shepherd-Banigan, K. P. Decosimo, J. Dadolf, A. Choate, E. P. Mahanna, N. R. Sperber, V. Wang, K. A. Allen, S. N. Hastings, C. H. Van Houtven

**Affiliations:** 1 Center of Innovation to Accelerate Discovery and Practice Transformation (ADAPT), Durham Veterans Affairs Health Care System, 508 Fulton Street (Mailcode 152), Durham, NC 27705 USA; 2 Department of Population Health Sciences, School of Medicine, Duke University, 215 Morris Street, Durham, NC 27701 USA; 3 Sanford School of Public Policy, Duke University, Durham, NC 27710 USA; 4 Duke-Margolis Center for Health Policy, Duke University, Durham, NC 27710 USA; 5 Center for the Study of Aging and Human Development, Duke University School of Medicine, Durham, NC 27710 USA; 6 Department of Medicine, Duke University Medical Center, 300 Morris Street, Durham, NC 27701 USA; 7 Department of Medicine & Thurston Arthritis Research Center, University of North Carolina at Chapel Hill, Chapel Hill, NC 27599 USA; 8 Geriatrics Research, Education, and Clinical Center, Durham VA Health Care System, Durham, NC 27705 USA

**Keywords:** Caregiving, Aging, Implementation research, Health services research, Long term care

## Abstract

**Background:**

Caring for a growing aging population using existing long-term care resources while simultaneously supporting and educating family caregivers, is a public health challenge.

We describe the application of the Replicating Effective Programs (REP) framework, developed by the Centers for Disease Control Prevention and used in public health program implementation, to scale up an evidence-based family caregiver training intervention in the Veterans Affairs (VA) healthcare system.

**Methods:**

From 2018 to 2020, clinicians at eight VA medical centers received REP-guided implementation including facilitation, technical assistance, and implementation tools to deliver the training program. The project team used the REP framework to develop activities across four distinct phases – (1) pre-conditions, (2) pre-implementation, (3) implementation, and (4) maintenance and evolution – **a**nd systematically tracked implementation facilitators, barriers, and adaptations.

**Results:**

Within the REP framework, results describe how each medical center adapted implementation approaches to fit local needs. We highlight examples of how sites balanced adaptations and intervention fidelity.

**Conclusions:**

The REP framework shows promise for national expansion of the caregiver training intervention, including to non-VA systems of care, because it allows sites to adapt while maintaining intervention fidelity.

**Trial registration:**

NCT03474380. Date registered: March 22, 2018.

## Background

Unpaid family and friends (“family caregivers”) who assist adults with caregiving needs report insufficient training to perform their role [1, 2]. Additional challenges for family caregivers include reduced work hours and lost income [3, 4], which contribute to high rates of caregiver burden, depression, and other health detriments [5–7]. Strengthening caregiver training and support could mitigate negative consequences of caregiving while increasing safety at home through higher quality of care from caregivers [8–11]. Better care in communities can avoid unnecessary or undesired acute hospitalizations, delay nursing home entry, reduce overall health care costs, and align care with patient preferences [12] which are priorities for health systems and clinical care teams alike.

Increasingly, there is recognition that family caregivers are an essential part of long-term care services and supports [13, 14]. In 2020, an estimated 41.8 million US adults are caregivers for a care recipient age 50 and older -- up from 34.2 million in 2015 [15]. Furthermore, there are 5.5 million family caregivers caring for former or current military personnel [16, 17]. The National Academies recommends that Veterans Affairs (VA) health system along with Centers for Medicare and Medicaid Services proactively identify and support family caregivers’ needs [9]. Effective interventions for caregivers are rarely implemented beyond local areas due to lack of funding supporting implementation beyond time-limited research studies and need to balance site-specific adaptation with fidelity to original program models [18, 19].

VA offers support to family caregivers through its National Caregiver Support Program -- resources, education and support to informal caregivers of Veterans through local VA health systems -- and through other caregiver support services developed across multiple service lines such as geriatrics services. This established infrastructure makes VA an ideal place to expand delivery of caregiver interventions [20, 21]. iHI-FIVES *(implementation of Helping Invested Families Improve Veteran Experiences Study)* is an evidence-based program designed to promote function and independence of Veterans through caregiver skills training. This program was designed and tested previously showing that it caused sustained improvements in caregivers’ and patients’ experience of VA care but did not increase patients’ days at home, change healthcare costs or reduce caregiver depressive symptoms [22]. The training targets caregivers of Veterans with recent referral to home- and community-based services, which signal an increase in needs and changes in types of care required at home. Core components of iHI-FIVES are a series of four classes [23] to help caregivers build self-care, health system navigation, and hands-on skills. Adaptable components include delivery staff (e.g., by service line), mode of delivery (e.g., in-person/virtual), optional content (six topical videos/phone scripts), and post-training “booster” calls. Scaling up evidence-based caregiver support interventions, while tailoring to site-specific needs regarding instruction space, facilitator staffing models (e.g., social workers only vs. with nurses or psychologists, and within or across service lines), degree of need, and funding, may be an approach to address caregiver support gaps. We describe the application of the Replicating Effective Programs (REP) framework to scale up an evidence-based family caregiver training intervention in the VA.

## Methods

### Overview of iHI-FIVES multi-site expansion

Based on initial results of the HI-FIVES program [22], VA National Caregiver Support Program advocated for its expansion. The impact of HI-FIVES on clinical outcomes for caregivers and their older Veteran care recipients has already been established. What is missing is additional testing regarding implementation strategies to support scaling and sustainment -- a type III effectiveness-implementation design is needed. Through the Optimizing Function and Independence Quality Enhancement Research Initiative (Function QUERI) program’s partnership between VA local and national clinical leaders, a facilitated rollout of the HI-FIVES program was launched across eight participating VA medical centers, using a hybrid type III effectiveness-implementation design, from 2018 to 2020 [24]. The Function QUERI program is funded by QUERI, national VA program leveraging scientifically-supported quality improvement methods, coupled with Veterans’ preferences and needs, to implement evidence-based practices rapidly across VA systems of care [25]. QUERI’s mission includes 1) rapid translation of research findings and evidence-based treatments into practice; 2) increasing research impact through partnerships and rigorous evaluation; and 3) promoting implementation science and the VA as a learning healthcare system.

### Site recruitment

To recruit sites, Function QUERI presented on national calls to VA providers in caregiver support and geriatric services. Function QUERI followed up with informational calls to interested sites and recruited VA medical centers that signed a participation agreement indicating leadership support and willingness to be randomized to specific start dates. Whereas each site had flexibility on which family caregivers to recruit, all agreed to include caregivers of Veterans who had received a referral in the past 3 months to five home- and community-based services: homemaker home health aide services; home-based primary care; respite care; adult day health care; and Veteran-directed care.

### Implementation framework: replicating effective programs

Function QUERI’s expansion of iHI-FIVES used a systematic implementation framework called Replicating Effective Programs (REP), a strategy framework that facilitates implementation across all its phases. Originally developed as a public health framework to support uptake of HIV behavioral and treatment interventions in community settings, REP standardizes implementation activities across four phases: 1) Pre-Conditions (identify needs, site-specific conditions, and barriers); 2) Pre-Implementation (develop technical assistance, identify champions, hold orientation meetings and train staff); 3) Implementation (provide technical assistance and measure intervention fidelity); and 4) Maintenance and Evolution (revise practices to facilitate adoption and prepare for program sustainability) [26]. REP has been empirically tested and validated through randomized controlled trials and shown to be effective in promoting uptake and fidelity of clinical interventions in various healthcare organizations including in the dissemination of a VA transitional care intervention to support high-risk patients and their family caregivers [27, 28]. Function QUERI chose REP because it specifies core elements and operationalizes those elements to local structure.

REP was used to guide facilitation, technical assistance, and implementation tools to support program implementation of iHI-FIVES and to address a major barrier to program adoption identified by operational partners: *limited clinician and other human resources*. REP enables sites to tailor programs to maximize use of existing personnel. It helps break down this global barrier of limited clinician and other human resources by providing guidance on program core components while promoting flexibility and potential adaptation so that sites can select approaches that best fit local conditions and resources [24]. REP is pragmatic in emphasizing user-friendly, low-burden implementation packages for end users for large-scale rollouts with relatively low need for additional local implementation resources [29]. A modified REP model has been successfully used in the dissemination of a VA transitional care intervention to support high-risk patients and their family caregivers [28].

Specific methods for data collection during this REP-guided process included the following. Each of the five REP pre-implementation calls and post-implementation activity report calls followed a structured format of topics appropriate to the stage of implementation. A call notes template was developed for each call based on that call’s agenda and included call topics, call participants and roles, and call length. A study team member (implementation specialist or research assistant) familiar with REP call content took verbatim notes, with individual speakers documented; the notes were reviewed after the call by the call facilitator and the project manager for accuracy and completeness. Given that there were often multiple sets of notes recorded by the research team, one set of notes was then compiled and used as the official copy. Action items and key observations were derived from the call notes and sent to team members who had participated in the call for “member checking” – a form of participant validation to explore credibility of results [30]. The call facilitator and notetaker reviewed the previous call’s notes before each ensuing call. The finalized call notes were incorporated into a searchable master notes document to facilitate recall of key decisions and action items across the period of REP site facilitation. One team member also periodically extracted key REP adaptation observations (adaptations, barriers, facilitators) and entered them into a standardized REP tracking matrix. At the completion of all site REP calls, Function QUERI implementation specialists reviewed the entire set of call notes and did a final extraction of information about site adaptations and entered the information into the REP tracking matrix.

## Results

**Fig. 1:**
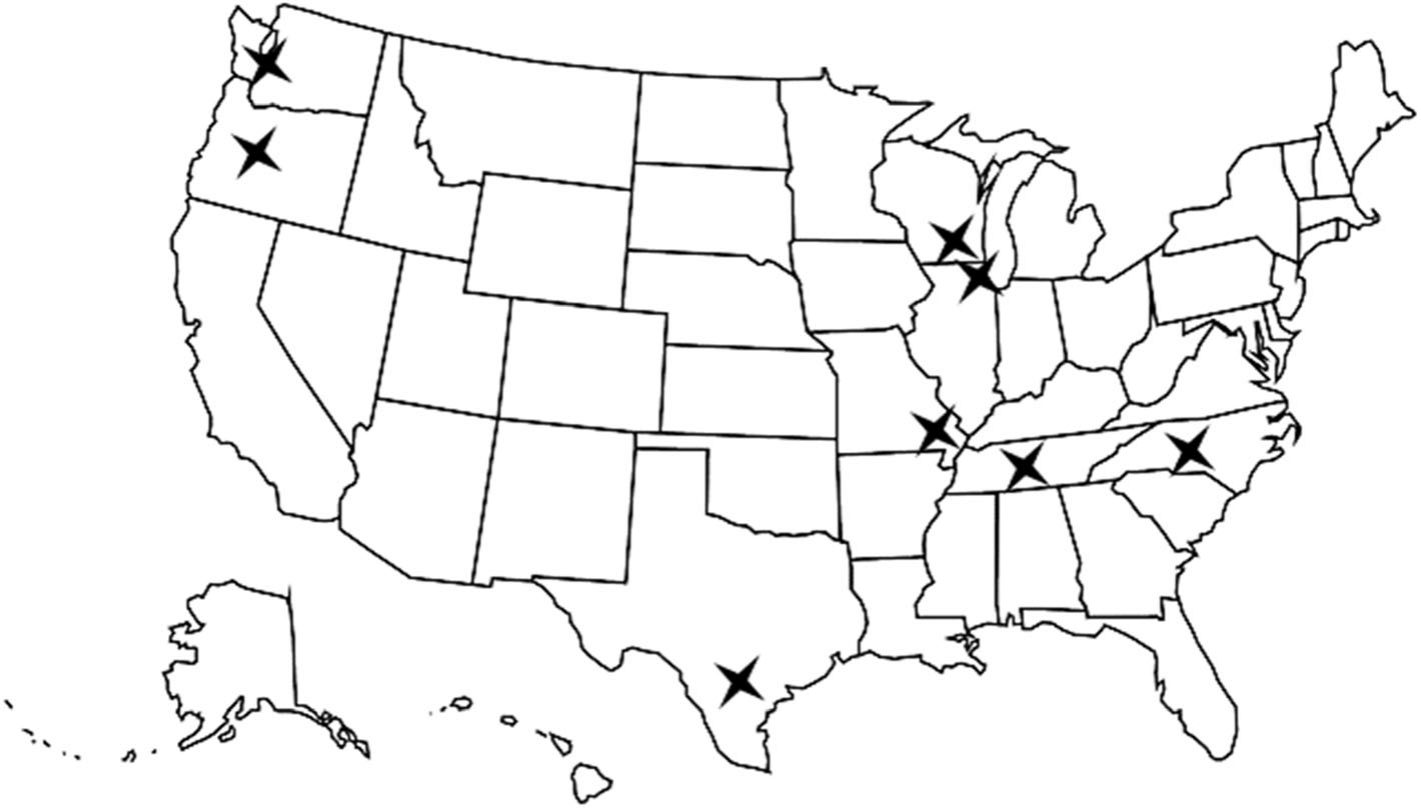
Participating Sites

Eight VA sites agreed to implement the iHI-FIVES program in the 2 year study (Fig. 1). Sites received approximately 4 months of technical support over the course of implementation. Table 1 details characteristics of eight currently participating sites and summarizes all of the Covid-19 adaptations made; all sites began delivering the curriculum virtually in April 2020. Audio line only is being used for some sites rather than video delivery as there were concerns from caregivers about confidentiality. Prior to Covid-19, caregiver trainings were delivered in-person with two sites incorporating video conferencing or phone session. All sites included a VA medical center as a base for program delivery. Three sites also used outlying VA outpatient clinics, and one site collaborated with community organizations to hold trainings. Two implementation specialists (MPH and MSW-trained) from the research team served as content and implementation support experts who provided training and mentoring across sites while research assistants handled notetaking at meetings.

**Table 1:** Site Context and Adaptations per Site (*n* = 8)

	Site A	Site B	Site C	Site D	Site E	Site F	Site G	Site H
**Hospital complexity**^**a**^	1A	3	1A	1A	1A	1A	1B	1A
**Relative Star Rating**^**b**^ 2018 (1–5)	2	3	3	3	3	2	5	3

**Target** **audience** *Caregivers of Veterans referred to the following services*	• Bowel and Bladder care caregiver stipend • Home and Community-based consults (HCBS)	• Respite Care • Caregiver Support Program (CSP) rosters	• Bowel and Bladder stipend • HCBS consults	• Transitional Care • Caring for Older Adults and Caregivers at Home (COACH) • HCBS consults	• Vision Impairment consults • HCBS consults	• Geriatric clinic rosters • Neurology clinic rosters • HCBS consults	• HCBS consults	• HCBS consults
**Delivery team service lines** *Comprised of social workers, nurses, and psychologists*	• Transition Case Management • Caregiver Support Program	• Caregiver Support Program	• Geriatrics • Social Work service (spinal cord injury, Transitional Case Management) • Psychology • Caregiver Support Program	• Caregiver Support Program (CSP)	• Social Work service (Primary care, geriatrics, palliative care spinal cord injury, visual impairment) • Caregiver Support Program	• Geriatrics • Social Work service (neurology) • Caregiver Support Program	• Social Work service (primary care, purchased care) • Psychology • Occupational Therapy • Caregiver Support Program	• Social Work service (inpatient, neurology, primary care) • Chaplaincy • Caregiver Support Program
**Optional content**	Videos, in-person booster sessions after training		Videos					
**Selected mode of delivery**	In-person weekly	In-person + video conference, 1-day training	In-person weekly	In-person weekly + 2-day training	In-person weekly + 2-day training	In-person weekly + remote video or teleconference weekly	In-person weekly	In-person weekly
**Training location**	VA Medical Center	Chamber of Commerce, VFW, VA Medical Center	VA Medical Center	Outlying VA outpatient clinics, VA dialysis clinic, VA Medical Center	Outlying VA outpatient clinics, VA Medical Center	VA Medical Center	Outlying VA outpatient clinics	Outlying VA outpatient clinics, VA Medical Center
**Covid-19 adaptations**	• Conference call group trainings	• Conference call group trainings	• Telehealth group trainings	• Conference call group trainings	• Telehealth group trainings	• No adaptations already offering remote options	• Conference call group trainings	• Conference call group trainings

^a^Complexity level: A rating that divides Veteran’s health facilities (VA) facilities into five levels (1A, 1B, 1C, 2, 3, highest to lowest), based on levels of patient volume and risk, teaching and research, intensive care units, and physician specialist staffing. ^b^ Star rating: A composite indicator of hospital performance relative to other VA medical centers (scale 1 to 5; low to high)

The *Pre-Conditions Phase* of REP includes identifying a need for the intervention and what will make it effective for the local setting, identifying a local champion, packaging the intervention for training, and identifying barriers [27, 28]. Site recruitment occurred in the pre-conditions phase, whereby enrolled sites expressed interest in joining the research study because they had identified the need to address gaps in caregiving training and support. At that time, Function QUERI (also called ‘the research team’) advised sites on strategies to secure buy-in from their medical center leadership. The participating site’s implementation team was responsible for activating leadership support, collaborations between clinical services, and participation across professional disciplines. The implementation team also assessed local resources and capabilities, as well as potential barriers to delivering iHI-FIVES. The research team worked closely with the local champion to articulate the site-specific goals of the program and to identify expected resources needed for implementation. For example, one site (Site E, Table 1) identified shortages in staffing in their Caregiver Support Program.

The research team invited executive leadership teams to site calls. In addition, the research team provided talking points for provided site implementation teams to share directly with their leadership quarterly meetings; reports back to the facility from the research team showing specific metrics assisted this process as well. As part of REP, the research team provided site implementation teams with marketing materials that they could use to share with other service lines, including fact sheets about the program, a health provider brochure, and a presentation template to adapt and use to spread the word within their VA medical center.

The *Pre-Implementation Phase* focused on collaborative preparation for implementation of the new program, including building facility-wide buy-in, further defining the implementation team, reviewing implementation support tools, staff trainings, and adapting the program to fit local contexts. For this phase, the research team worked with the local champion to identify the delivery team and to engage key stakeholders and decision-makers (e.g., program managers, service line chiefs, and front-line staff) involved in providing caregiver support. For example, recalling that Site E had identified staff shortages in one service line, in this phase they contacted other service lines to find trainers.

Over 3 months, the research team facilitated a series of team planning calls (Table 2) describing the program package: core components of iHI-FIVES (four in-person group classes delivered at least two times within a 6 month period), program materials (caregiver workbook, facilitator’s guide), optional content (six topical videos or phone scripts and post-training “booster” calls) and processes (delivery team composition and mode of delivery) [31]. The research team also provided technical assistance to tailor the program for local conditions and to avert potential implementation barriers (e.g., leadership support, training space, recruitment, and staff time). The research team helped trouble shoot challenges facing the implementation team as well. Technical assistance also included providing a recruitment tool that listed all Veterans referred in the past 3 months to the qualifying home- and community-based services, as well as modifiable electronic health record templates that provided sites a standardized approach to track program enrollment, attendance, and caregiver-reported outcomes such as depression (PHQ-2) [32] and caregiving burden (Zarit 4-item) [33].

**Table 2:** Topics for facilitated calls and site visit for iHI-FIVES (REP Pre-Implementation Phase)

Activity	Objective	Agenda Items
1) Welcome Call (1 h)	To understand purpose and components of Function QUERI and iHI-FIVES.	• Program goals, core and adaptable components, intended target audience • Resources needed for implementation
2) Curriculum Call (1 h)	To review iHI-FIVES training intervention.	• Clinical package and materials, core and adaptable components, delivery model options
3) Recruitment and Documentation Call (1 h)	To define iHI-FIVES participant recruitment and tracking documentation process and reporting.	• Identifying caregivers for recruitment, tools for documenting program delivery and outcomes • Discussing site-level processes for providing psychoeducational programs and adapting documentation
4) Marketing Call (1 h)	To tailor iHI-FIVES marketing materials and present ideas to increase recruitment and the impact of iHI-FIVES.	• Standardized tools and materials for marketing program internally for stakeholder buy-in and externally to Veterans and caregivers • How to adapt materials for target audiences and developing plans to address potential recruitment barriers
5) Site Visit Planning Call (1 h)	To discuss and finalize upcoming site visit activities.	• Planning of the upcoming site visit activities, presentations, technical assistance meetings • Identifying staff for train-the-trainer session
Site Visit (1–2 days)	To provide on-site technical assistance to assist with strategic planning, implementation, and potential barriers.	• Meet with delivery team, leadership, and internal stakeholders to discuss importance of caregiver support and the implications of iHI-FIVES to their site • Tour training facility. • Rapport and relationship building • Conduct train-the-trainer session on curriculum • Strategize and problem solve identified barriers or obstacles to successful launch
Optional Pre-Launch Call (30 min)	To provide technical assistance around any barriers to program launch	• Discuss barriers and potential solutions to program launch (e.g., recruitment, space, etc.)

Lastly, for pre-implementation, the research team conducted a site visit to understand local context (e.g., classroom space, parking, facility navigation) and help sites build momentum for program launch. Site visits included working with local champions to present the iHI-FIVES program to medical center leadership and a meeting with the implementation team to develop strategies to further tailor the program, address remaining barriers, and answer questions. Regarding leadership engagement, the research team met with medical centers’ leadership to talk about the program and its efficacy across a number of different domains such as numbers of caregivers served, satisfaction with current supports, and considerations for efficiency. These meetings were also attended by points of contacts where the research team could give them recognition in front of leadership for their commitment to training caregivers of Veterans. The REP call notes showed that sites especially valued the research team communicating with their leadership about their participation in the study and the consequent expansion of caregiver training at their site.

Also during the site visit the site visit the research team provided a two-and-a-half-hour train-the-trainer session for identified delivery staff that provided a detailed review of the curriculum, program marketing templates, and a discussion of best practices for delivery. Debriefing with points of contact at the site visits indicated that many felt the site visit gave them confidence to launch by answering many of their remaining questions about the training program features and processes such as marketing and recruitment. At this point, sites were able to launch their programs.

Program launch occurs in the *Implementation Phase*, which is defined as sites starting their first round of the four iHI-FIVES training classes within the randomized launch window. As sites implemented their first round of training, the research team provided ongoing technical assistance and facilitation around adaptations, barriers to delivery, increasing buy-in, and ensuring intervention fidelity (that is, fidelity to key training program components). During these calls, implementation teams also shared how well the training was received by delivery staff and the participating family caregivers. The research team then presented an activity report on the first round of training, with information from the electronic health record templates regarding program implementation outcomes such as reach (number of caregivers offered training), attendance, characteristics of the target audience (age, gender), caregiver measures (depression PHQ-2 and burden Zarit 4-item), and class evaluation summaries. After report review, Function QUERI facilitated troubleshooting additional implementation barriers. For example, if reports showed low caregiver attendance, Function QUERI and the local implementation team discussed potential strategies to increase attendance (e.g., marketing, virtual options, and varying training time or location).

The final REP phase is *Maintenance and Evolution* and included ongoing technical assistance with implementation teams to further refine program adaptations and clinical package. As part of this phase, Function QUERI continued to provide facilitated calls after each round of training with ongoing presentation of activity reports. These calls included review of key program activities and metrics, intervention fidelity, and caregiver perception of program value via evaluation summaries. These reports were valuable in illustrating program impact with facility leadership to garner continued support and make the case for sustainability. For example, Site A (Table 1) used the report and infographic of impact to share with leadership and to leverage support from their local public affairs office.

Assessing the data and detailed notes in the REP tracking matrix as sites reached the *Maintenance and Evolution* phase, the data illustrated that all sites adapted some features of the program to meet their needs. As far as target audience, six of the eight sites added additional sources for caregiver recruitment beyond home and community-based consults (Table 1). Six of the eight sites chose a delivery model that used more than one clinical service line; of these, four of the six sites used three or more service lines to build the delivery team (Sites C, F, G, H) while two sites used two service lines. Three of the eight sites adapted the weekly delivery of classes by stacking them either all on 1 day or over 2 days. To meet the need for caregivers who could not leave their Veteran at home, one site (Site C) offered activities for Veterans on-site while the caregivers attended the trainings. Two sites incorporated remote delivery from the start of the program, prior to the pandemic, in order to better meet the needs of their sites caregivers. Notably only two of the six sites incorporated any optional materials (i.e., videos of topics or post-training “booster” calls) (Table 1).

The REP tracking matrix showed that fidelity to the intervention was generally high across all sites; they succeeded in delivering the agreed upon content and using agreed upon modes of delivery, that is, offering the core curriculum, and using agreed upon delivery teams (e.g., facilitators from within service-line or across service-lines). The Covid-19 pandemic required sites to pivot to virtual delivery at a time when the pandemic placed increased clinical demands for their time. Whereas all sites successfully pivoted to virtual delivery, challenges with Covid-19 resulted in one missed training for two sites and caregivers’ ability to participate was moderately hampered at the two newest sites that started in March 2020 (Table 1). To support program sustainability and spread, we have implemented quarterly “Diffusion Network” conference calls. These calls serve as an opportunity for our partners to discuss implementation challenges and obstacles, as well as to share innovation and ingenuity in delivering support programs at their sites. As Covid-19 abruptly challenged our sites to quickly pivot an in-person delivery to a virtual or telephone delivery, we encouraged our partners to come together to strategize and problem solve. During these calls the group provides each other with tips and strategies for classroom management and recruiting, as well as sharing the positive outcomes they witnessed during this challenging time. Although not a component of REP, this “Diffusion Network” conference call has served as an additional source of program support for the partners engaged in program delivery at their sites.

## Discussion

Function QUERI used the REP framework as a strategy to guide implementation activities and organize them by phase. The REP framework allows for capitalizing on local resources available to programs. The REP framework was also used to elicit allowable program adaptations and track program adaptations made across sites. Based on our experiences across an integrated healthcare system we share four key lessons learned: (1) all sites adapted the training program to optimize delivery at their site, (2) sites valued the site-visit but varied in the level of facilitation they needed to launch, (3) peer mentoring arose as an unexpected but valuable resource to sites, and (4) facilitation by the Function QUERI team successfully helped sites surmount many but not all of the implementation barriers faced.

### All sites adapted the training program to optimize delivery at their site

Through activities and facilitation, local implementation teams had dedicated time to tailor the program across multiple domains. These domains included adjustments to the intended iHI-FIVES target audiences, the delivery model, and whether to include optional topics or materials. All sites made adaptations to maximize use of existing resources to support program implementation, while maintaining intervention fidelity. For example, sites were told on facilitation calls that training ideally should be delivered as one class per week for 4 weeks, while also being told that they had latitude in delivery based on their site’s needs. Thus, stacking the classes over a shorter time period both made it easier for rural caregivers to attend and was consistent with an allowed intervention adaptation. Staff time was a major constraint for sites, and the low uptake of optional materials reflects the time constraints the implementation team expressed facing. Time constraints appeared in the REP tracking matrix data as an important theme. Lack of staff time overall was the reason sites gave for not using these optional materials. Overall, allowing flexibility using the REP framework helped maintain fidelity to the intervention with latitude for site-specific constraints.

### Sites valued the site-visit but varied in the level of facilitation they needed to launch

Function QUERI determined that it was helpful to provide technical support at regular checkpoints within the REP phases – specifically, Pre-implementation and Implementation phases -- to help teams address key implementation barriers and to discuss the balance between those challenges and fidelity to the core program components (e.g., fidelity to the intervention). As described in the Results section, Function QUERI provided external facilitated [34] support through five planning calls and developed standardized tools to support implementation such as electronic health record templates to track program activities and marketing templates to help sites recruit caregivers. At the site visit, the “train-the-trainer” sessions delivered by Function QUERI provided an additional interactive space for training facilitators to communicate the curriculum components, discuss facilitation techniques, training logistics, and strategize approaches to solve site-specific challenges depending on their selected mode of delivery and target audience. The site visit helped Function QUERI build rapport and trust among the local implementation teams, which enhanced consequent technical assistance in reviewing activity reports and interactive problem solving.

All sites received approximately the same number of calls and amount of technical assistance. And yet, some sites emailed with questions frequently, while others engaged with Function QUERI only at scheduled times, declining the optional pre-launch call. Function QUERI found ongoing technical assistance in the REP Maintenance and Evolution Phase particularly useful when facing challenges such as unexpected reductions in staffing. The research team would strategize solutions that allowed them to remain adherent to the study agreement (e.g., delivery of two rounds of iHI-FIVES each 6 months). That said, not every site reported challenges with launch or initial sustainment. The variation in available resources across sites – available staff to devote to the program, available funds to cover additional staff time, available VA space which is normally tight -- is important to keep in mind in considering national implementation, because individual facilitation in the REP framework is time-intensive for both the research team and the site’s implementation team. Tailoring the dose of implementation activities in future efforts could be a more efficient use of resources, such as reducing number of contacts with those sites that express a desire to receive less support. This tailoring could be guided by existing adaptive implementation strategies which include evaluation after each phase of roll-out to determine what the client needs and how that changes the approach to the next phase or implementing a more iterative approach where sites need to return to a prior phase for deeper learning or coaching [35, 36].

### Peer mentoring arose as an unexpected but valuable resource to sites

In early REP phases, information exchange was primarily between Function QUERI and the local implementation team, then in later phases, specifically the *Maintenance and Evolution* phase, the sites wanted to connect directly with one another to share information. With permission, Function QUERI connected implementation sites to each other to ask questions and discuss challenges. The demand for peer support has been operationalized further into “diffusion network” calls where lessons learned can be shared among all iHI-FIVES sites along with general idea-sharing and collegial support.

### Facilitation by the function QUERI team successfully helped sites surmount many but not all of the implementation barriers

Function QUERI’s strategy for using REP was effective in collaborating with sites to identify potential barriers, particularly in the *Pre-Conditions* phase We observed that teams could overcome system constraints as they implemented the evidence-based program. Teams found unique solutions to their site-specific constraints. For example, space at some VA medical centers was limited, especially for programs not explicitly focused on Veteran care, and teams made adaptations to this by modifying training schedules and utilizing off-site space. Also, caregivers who were not be able to leave the Veteran alone at home had options with on-site care for the Veterans offered at one site. These solutions helped surmount barriers while maintaining fidelity to core components of the iHI-FIVES curriculum.

However, some site-specific barriers could not be surmounted through facilitation from the Function QUERI team. For example, caregiver schedule constraints are great and often unpredictable posing a challenge of maintaining a quorum for trainings, even when on-site care for the Veteran was offered. In addition, each site has its own organizational culture even though all are part of the larger VA system. Sites implementing iHI-FIVES identified challenges with organizational culture that will need to be addressed to improve program impact and sustainability. “The caregiver is not my patient, the Veteran is” was a paraphrase heard by some VA implementation teams; indeed, this may be a widely held opinion across many systems of care where the patient is the defined unit of care in practice and for insurance reimbursement. Providers may be unaware of caregiver support resources, misinterpreting the scope and availability of services. Engaging leadership at the medical centers to endorse the importance of supporting caregivers could facilitate cultural transformation to a more caregiver friendly VA. Additionally, some VA providers cannot get the same clinical credit for time spent with caregivers as they do with Veterans. Instituting a culture change lies largely outside of the research team. Changing incentives broadly to support caregivers across the VA health system could help achieve culture change.

As of December 2020, all eight sites were delivering virtually due to Covid-19, and sites are awaiting guidance from their medical center for when they can resume in-person trainings. Virtual trainings have been well-received by some caregivers as it makes it easier to attend, though reminder calls have been needed. The Diffusion Network calls also assisted this process by providing a forum for Covid-19 adaptations discussions among project teams. Other challenges include technical difficulties related to internet connections or capacity issues on conference lines, staff having limited availability to deliver the training due to having to cover essential clinical duties, and perceived lack of human connectedness as a barrier to caregivers being candid about their experiences. Another issue faced by facilitators was managing classroom dynamics remotely. It is challenging to manage someone who talks too much or too little, or strays off topic. When this is happening on a conference call the challenge is magnified. Solutions to the above have included instituting smaller online groups for intimacy, development of approaches to respond to caregiver distress remotely, and building in more time for instructor preparation and online class time. Whereas the research team could brain storm solutions with sites, ultimately, the sites had to find tailored solutions to surmount these and other unexpected training barriers, such as those posed by Covid-19.

## Conclusions

The role of Function QUERI is to rapidly translate research findings into practice, and REP is a practical framework for helping sites implement in a standardized way and yet with the ability to adapt to their own resources and contexts. REP offers an accessible approach with proven success to scale up evidence-based caregiver support programs which can augment the work of clinical teams [28]. Importantly, using the REP framework with facilitation with individual sites has been well received and all eight sites were able to launch their training in a timely fashion. The lessons learned show the evolution, challenges, and solutions that implementation teams experienced, which may be helpful to others considering implementing evidence-based programs in their health system. Using standardized approaches to track all adaptations in a matrix allowed us to form a complete picture of adaptations made at all participating sites. Other teams may find use of similar methods in recording site-level adaptations valuable, in order to understand fidelity to core program components at study end.

Virtual trainings may help more caregivers access training than were able to in the original randomized controlled trial requiring in-person attendance. Schedule burdens, health burdens, and reticence to ask for help are barriers for caregivers seeking caregiver training and support despite high-perceived need for information and desire for help with caregiving [37–39]. Thus, innovations such as adding virtual options for rural sites and stacking classes on fewer days allows more family caregivers to engage in the training. The transition to virtual training due to Covid-19 has been negotiated well by each site. Overall, sites made adaptations that allowed them to maintain fidelity to core program components, while innovating processes so they could be successful. This paper focused on the use of REP to implement an intervention; we will report implementation outcomes, caregiver outcomes, and patient outcomes in a future manuscript since the two-year study ended recently in October 2020.

Synthesizing implementation barriers and facilitators across sites, Function QUERI developed a step-by-step implementation guide using REP phases on how to plan for, deliver, document, and sustain the iHI-FIVES training program to promote further uptake of iHI-FIVES among VA medical centers. In addition, the diffusion network was established to help current sites with sustainability and self-sufficiency. From the positive experience incorporating “Diffusion Network” calls, these calls may be a thoughtful addition to stages of a REP approach for other projects. Importantly, the diffusion network and implementation guide require fewer Function QUERI resources -- necessary for national program feasibility. Examining sustainability will be important future research of the Function QUERI team.

## Data Availability

Not applicable.
